# Unraveling Hidden Order in the Dynamics of Developed and Emerging Markets

**DOI:** 10.1371/journal.pone.0112427

**Published:** 2014-11-10

**Authors:** Yonatan Berman, Yoash Shapira, Eshel Ben-Jacob

**Affiliations:** School of Physics and Astronomy, The Raymond and Beverly Sackler Faculty of Exact Sciences, Tel-Aviv University, Tel-Aviv 69978, Israel; University of Maribor, Slovenia

## Abstract

The characterization of asset price returns is an important subject in modern finance. Traditionally, the dynamics of stock returns are assumed to lack any temporal order. Here we present an analysis of the autocovariance of stock market indices and unravel temporal order in several major stock markets. We also demonstrate a fundamental difference between developed and emerging markets in the past decade - emerging markets are marked by positive order in contrast to developed markets whose dynamics are marked by weakly negative order. In addition, the reaction to financial crises was found to be reversed among developed and emerging markets, presenting large positive/negative autocovariance spikes following the onset of these crises. Notably, the Chinese market shows neutral or no order while being regarded as an emerging market. These findings show that despite the coupling between international markets and global trading, major differences exist between different markets, and demonstrate that the autocovariance of markets is correlated with their stability, as well as with their state of development.

## Introduction

In many models of financial markets, the statistical properties of the price change are crucial factors. For example, these statistical properties are widely used in derivative pricing, where certain types of stochastic behavior of the asset price are taken as underlying assumptions. Many stochastic models of stock market price dynamics use Brownian motion like processes, where the price return is randomly generated from a distribution that is derived from empirical data [1]–[5]. In most of these models, the distribution is normal, a truncated Lévy flight or other heavy tailed distributions [2], [3], [5]–[8]. This approach is supported in part, by the observation that price changes in financial markets have practically no memory, and the autocorrelation or autocovariance functions of the price change have a characteristic time scale of a few trading minutes [9], [10]. Moreover, the assumption of stock returns randomness suggests that additional statistical properties of the return are inforecastable, such as its volatility. However, it was already shown that some temporal order exists in the variance of the market volatility [11]. Some predictability of stock and index prices was also found for various markets in certain periods of time [12]–[16]. Most of these studies use statistical tests to refute the random walk hypothesis [12], [17] for very specific time periods and indices, inspired by the works of Lo and MacKinlay [12], [15].

In addition, auto-regressive models were suggested as appropriate mathematical frameworks for modeling economic and financial processes with explicit autocorrelation [18]–[21]. These models were found successful in making predictions and providing explanations to various financial phenomena [18], [22]. However, they require a prior knowledge of the underlying autocorrelation. This undermines the success of these models.

While the overall random nature of financial markets is universal and does not depend, in theory, on specific market characteristics, in practice, different markets demonstrate a variety of behaviors. In the context of developed and emerging economies, several fundamental economic and financial differences are well known, such as growth rate, industrialization, financial sector size and stock market liquidity [23]–[26].

Here we demonstrate the existence of hidden order in the autocovariance of major stock indices and suggest that there exist a fundamental difference in terms of the autocovariance between developed and emerging markets that showed relatively high values of autocovariance during the past decade. Moreover, we provide a method, which will be referred to as the *Responsive Algorithm*, or *RA*, that can serve as a tool for identifying major deviations from a random behavior in market returns. The tools used to identify these deviations, are also used to demonstrate the qualitative differences between developed and emerging markets, in addition to analyzing differences between several European markets during the financial crises of the past decade.

## Results

Within the framework of this paper, the most important mathematical property that characterizes time series is the autocovariance within the relative change, or return, series. Given a time series representing an asset price, an index or an artificially produced random walk, 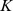 (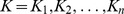), we define its relative difference series 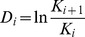 for 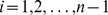. Using 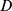 we can define the following two vectors: 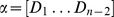 and 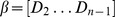. The autocovariance will be defined as:
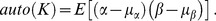
(1)


Notice that 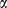 and 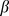 are dependent random variables and are very similar, so that Eq. (1) becomes 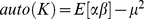. This definition introduces bias to the estimation of the autocovariance, due to the use of the sample means. This bias can be eliminated, and the unbiased expression for the autocovariance becomes:
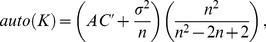
(2)where 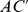 is the calculated sample autocovariance, 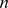 is the sample size and 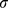 is the relative difference series sample standard deviation (see full details in the Methods section). Notice, in addition, that only the autocovariance for lag of one time step is taken into account.

Random walks are Markov stochastic processes and we would expect their autocovariance to be close to zero. Performing a numerical analysis of the autocovariance of random walks with different values of 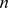 and 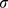 and using Eq. (2) showed that indeed, the average autocovariance does not depend on 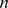, and is normally distributed around 0. It is also noteworthy that the average autocovariance is independent on the choice of 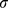. It is an important result, as it allows us not to take 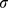 into consideration when trying to estimate the deviation of real market data from random walks. In addition, the form of Eq. (2) is likely to produce similar results even after omitting the assumption of normality of 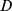. It is therefore implied that a considerable discrepancy between the autocovariance calculated for real market data and 0, is an indication of the data divergence from random behavior. Such divergence demonstrates the existence of temporal order in the autocovariance.

We now use sliding windows of different lengths, denoted as 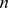, and calculate the average autocovariance of all windows for different market indices. In order to estimate the deviations of indices from random walks, the results are compared to the results of the same analysis done for shuffled data. The shuffled data was constructed by randomly permuting the relative differences time series of a given index, and rebuilding the index time series using the permuted series of relative differences. Shuffling preserves most of the statistical properties of the time series, while removing any temporal order. Hence, performing the analysis on shuffled data will eliminate deviations from a random walk that are associated with order, such as the autocovariance. The comparison between the results produced using shuffled and non-shuffled data might produce indications for the existence of such deviations and therefore, of order.

**Figure 1:**
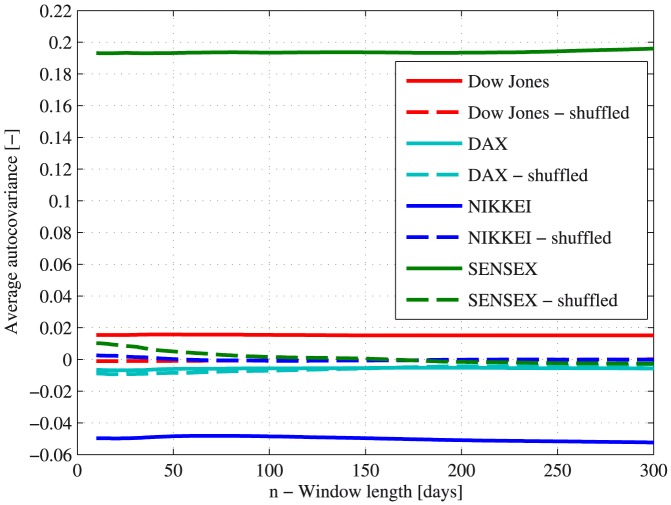
The average autocovariance in several major indices and their dependence on the sliding window length. The average autocovariance of several major indices is presented for real market data (red - Dow Jones; cyan - DAX; blue - NIKKEI; green - BSE SENSEX) and for the shuffled indices data (in dashed lines). The presented results are the outcome of an analysis taking account of all the available data for each of the indices: 1896–2013 for the Dow Jones index, 1990–2013 for the DAX index, 1986–2013 for the NIKKEI index and 1997–2013 for the BSE SENSEX index.

Following the described procedures, the autocovariance of several major stock market indices was analyzed. The results are presented in Fig. 1. Two major observations can be deduced following these results:

- The analyzed indices show average autocovariance that is above and below the random average autocovariance. The shuffled data produces results that are similar to a random walk, which provides an additional indication for the substantial deviation of the autocovariance from a random series. These results indicate the existence of temporal order in the indices return.
- The dependence of the average autocovariance on the value of 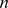, the sliding window length, is very weak. This implies that in order to properly analyze the autocovariance of stock market indices, it is sufficient to perform the analysis for sliding windows that are up to several trading months long.

Since the daily returns are not stationary processes, performing the analysis for very large sliding windows might distort its results. Following this, in addition to the weak dependence of the results on the sliding window length, only sliding windows of up to 100 days in length will be analyzed.

### The responsive algorithm

The temporal order found in the autocovariance of market indices returns, as depicted in Fig. 1, implies that some degree of predictability can be found in stock market indices. In addition, it suggests that an algorithm can be formulated, so that it will extract the hidden autocovariance found in the market. We therefore describe such an algorithm that can help to identify positive or negative autocovariance in stocks or indices, called the *responsive algorithm*, or *RA*.

The RA is simple - take no position while the asset price is increasing; once it decreases, close the position and take a short position as long as it decreases; once the asset price increases again, close the short position, take a long position with the wealth gained so far, and repeat the previous steps.

Given a certain asset (stock, index, option, futures contract, etc.) with price 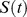, which depends on time 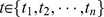 we apply the RA and produce 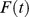, the investor's wealth, which is the sum of the total value of the investor's owned assets and the investor's wealth that is not invested in time 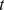. It is assumed that in time 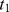 the investor owns one share (or one option, one contract, etc.) of the asset in discussion (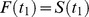). We also assume that 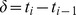, for any 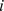, is a given positive time step. The *profitability ratio* is defined by 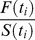, and provides the ratio between the wealth gained by using the RA and the buy-and-hold method. The definition of the profitability ratio obviates the need of adjusting the RA results due to interest and inflation, since these features effect the RA and buy-and-hold method similarly. Since our analysis of the autocovariance was done for a one day lag, we choose 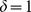 day for the RA, for all the performed calculations.

We would expect the RA to become more successful as the autocovariance of the asset price increases and less successful as it decreases. In order to test this assumption, we examined the results produced by the algorithm for random walks. The profitability ratio of the RA is found to increase exponentially with the autocovariance of the data series it is applied to. In addition, as the length of the series the algorithm is applied to increases, it is easier to make a distinction between the results for different values of average autocovariance. It can be concluded that there exists a positive correlation between the autocovariance found in asset price returns and the RA results, enabling its use in order to identify major deviations from zero-autocovariance or random data.

The RA resembles trend following investment strategies [16]. However, its aim is only to extract the hidden market autocovariance, and is not an actual investment strategy. It is also assumed, therefore, that no commission is paid for the different actions.

### The historical dynamics of autocovariance in the US stock market

The history of the autocovariance and its dynamics over the course of the 20th century is of great interest, especially for the US stock market, which demonstrates intriguing and inconsistent dynamics, with long periods of substantial positive average autocovariance among its 3 major indices - the Dow Jones, the S&P500 and the NASDAQ. In addition, while the autocovariance of the Dow Jones and the S&P500 indices behaves relatively similar, it differs considerably for the NASDAQ. These dynamics might indicate long term processes associated with the US stock market that made trading more intricate and efficient, and have characterized the maturing of major western markets.

**Figure 2:**
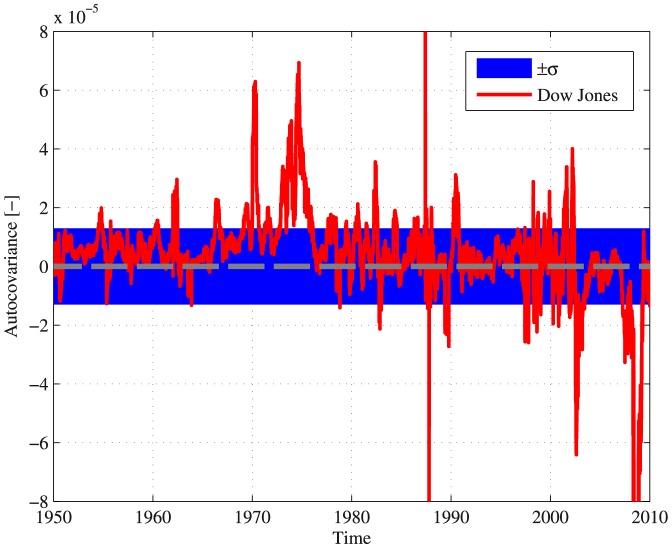
The autocovariance of the Dow Jones industrial Index 1950–2010. The calculations were made using a 100-day sliding window for the actual data (red). The blue area marks one standard deviation of the autocovariance from 0, estimated by data shuffling. Autocovariance 0 is marked by a dashed gray curve.

The autocovariance calculated for the Dow Jones index during the period 1950–2010 is presented in Fig. 2. The calculations were also performed for shuffled data, in order to estimate the deviation from random behavior. In addition, we used a statistical test similar to the Wald-Wolfowitz runs test [27], in order to quantify the statistical significance of the autocovariance deviation from a random behavior (see full details in the Methods section). The presented results indicate a transition at approximately 1980 - In the period 1950–1980 the autocovariance is found as consistently positive and significantly different from random (using the runs test, 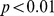). However, during 1980–2010, the results are much closer to random, and no statistical significance is found for the deviation of the results from randomness (using the runs test, 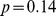). A more thorough analysis of these dynamics, which are closely related to the dynamics of trend following strategies [16], exceeds, however, the scope of this paper, and is left for future work.

### Emerging and developed markets

Emerging markets or economies are markets characterized by relatively large growth rates, demonstrating properties of both developing and developed countries [28], [29]. The term had been used extensively in the past few decades, and during the last decade it describes China and India, as the largest emerging markets, in addition to Russia, Mexico, Brazil, Turkey and others [29]. The results presented in Figs. 1 and 2 not only demonstrate the existence of temporal order in markets autocovariance, but also suggest that a major difference might exist between different markets at different stages. In order to test whether a fundamental difference exists between the autocovariance of developed and emerging markets, the analysis tools described previously were used. For the sake of proper comparison, the data taken into account was in the period 2000–2013 for all indices analyzed.

**Figure 3:**
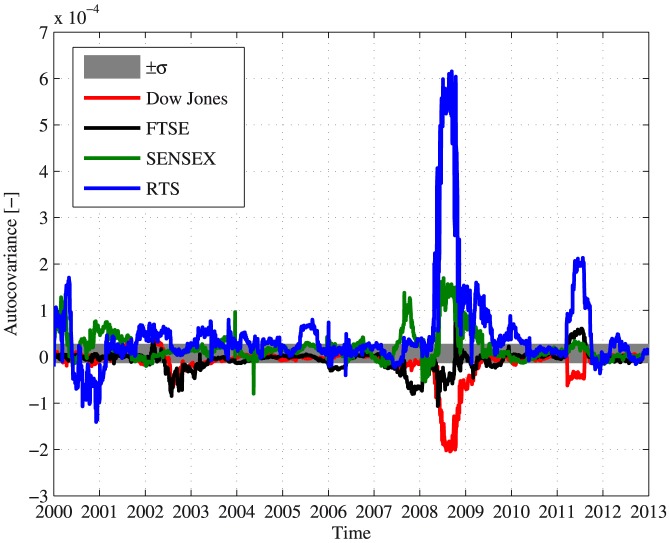
The autocovariance as calculated for major developed and emerging market indices during 2000–2013. The calculations were performed for the Dow Jones index (red), the FTSE 100 index (black), the Bombay stock exchange SENSEX index (green) and the Russian RTS index (blue), using a 100 days long sliding window. The gray area marks one standard deviation of the autocovariance from 0, estimated by data shuffling.

Fig. 3 presents the results of the autocovariance calculation using a 100 days long sliding window for the following major indices - the Dow Jones index, the FTSE 100 index, the Bombay stock exchange SENSEX index and the Russian RTS index. The calculations were performed for the daily closing value of each of the indices analyzed between January 1st 2000 and January 1st 2013. Data shuffling was also done for each of the indices. It was found that the autocovariance of the shuffled data was normally distributed around 0 with a standard deviation 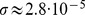.

The dynamics of the autocovariance found in various indices, as depicted in Fig. 3, indicate the existence of a qualitative difference between developed and emerging markets since 2000. The Dow Jones and the FTSE indices (in addition to the indices of other developed markets, such the DAX and the CAC indices, which were not presented in Fig. 3) behave qualitatively similar as both indices exhibit below zero autocovariance, most notably during 2007 and 2008. The RTS index and the SENSEX index, of the emerging markets of Russia and India presented an approximate reflection of the dynamics of the Dow Jones and FTSE indices and specifically exhibit above zero autocovariance during almost the whole period in discussion. In addition, the results imply that during the peak of the global financial crisis of 2007–2009, the indices demonstrate significant above/below zero autocovariance values (using the runs test, 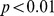). This result may indicate the existence of a herd behavior, which is stronger during crises and increases the correlations between single stocks in the market [10], [30]–[35].

**Figure 4:**
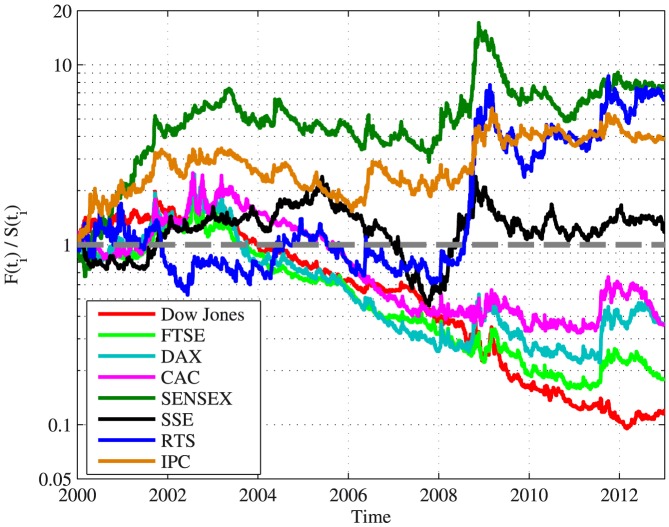
The RA results for various emerging and developed markets indices in the period 2000–2013. The ratio 1 is marked by a dashed gray curve. The results are presented for the American Dow Jones index (red), the FTSE 100 index (light green), the German DAX index (cyan), the French CAC index (magenta), the Indian SENSEX index (dark green), the Chinese SSE Composite index (black), the Russian RTS index (blue) and the Mexican IPC index (orange).

In order to further test whether a distinction between developed and emerging markets can be made, an analysis of the average autocovariance and the RA results was performed for the same period of time. The developed market indices taken into account were the Dow Jones index, the FTSE 100 index, the German DAX 30 index and the French CAC 40 index. The emerging market indices taken into account were the Indian SENSEX index, the Chinese SSE Composite index, the Russian RTS index and the Mexican IPC index. The results of this analysis are presented in Fig. 4. The average autocovariance values of the emerging markets were found to be significantly above 0 (using the runs test, 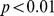), where for the developed markets they were below 0 (it can also be deduced from the results presented in Fig. 3). The average autocovariance of the SSE Composite index was very close to 0 and slightly above it, but with no statistical significance (using the runs test, 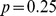).

These results, apart from indicating how these markets diverge from random series, suggest that the autocovariance of developed and emerging markets behaved fundamentally different in the past decade, indicating some characteristics of developed markets that the emerging markets lack and vice versa.

**Figure 5:**
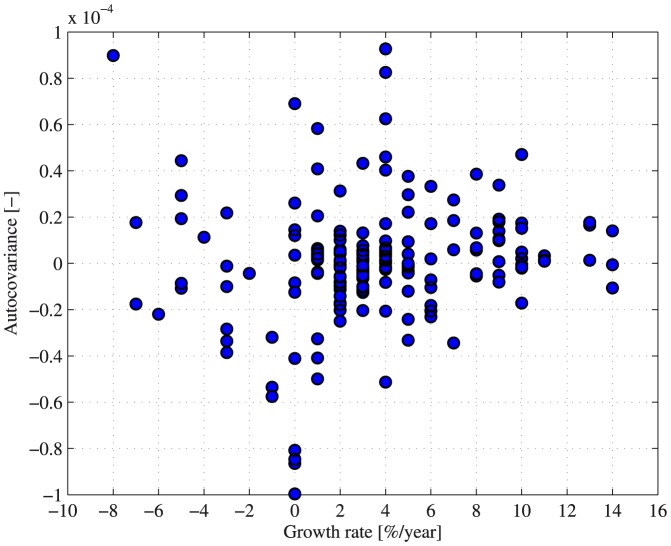
The autocovariance dependence on economic growth 2004–2012. The autocovariance of 21 stock market indices of 16 different economies (Brazil, China, Cyprus, France, Germany, Greece, India, Israel, Italy, Japan, Mexico, Portugal, Russia, Spain, UK and US) was calculated for each year between 2004 and 2012. In addition, the economic annual growth rate of each market was taken from the World Bank database [36]. The resulting scatter plot indicates that no significant relationship exists between these variables.

In order to test whether the high growth rates that characterize emerging markets are related to the high autocovariance observed in those markets, we calculated the autocovariance of 21 different stock indices in 16 countries (Brazil, China, Cyprus, France, Germany, Greece, India, Israel, Italy, Japan, Mexico, Portugal, Russia, Spain, UK and US) for each year during 2004–2012. The annual growth rate of each country was taken from the World Bank database [36]. The resulting scatter plot presented in Fig. 5 indicates that no significant relationship exists between these variables. The correlation between the calculated autocovariance and the growth rate was 0.06. These results imply that other characteristics of emerging markets are likely to produce the autocovariance nontrivial behavior. We will elaborate on the possible sources for the observed differences in the discussion.

**Figure 6:**
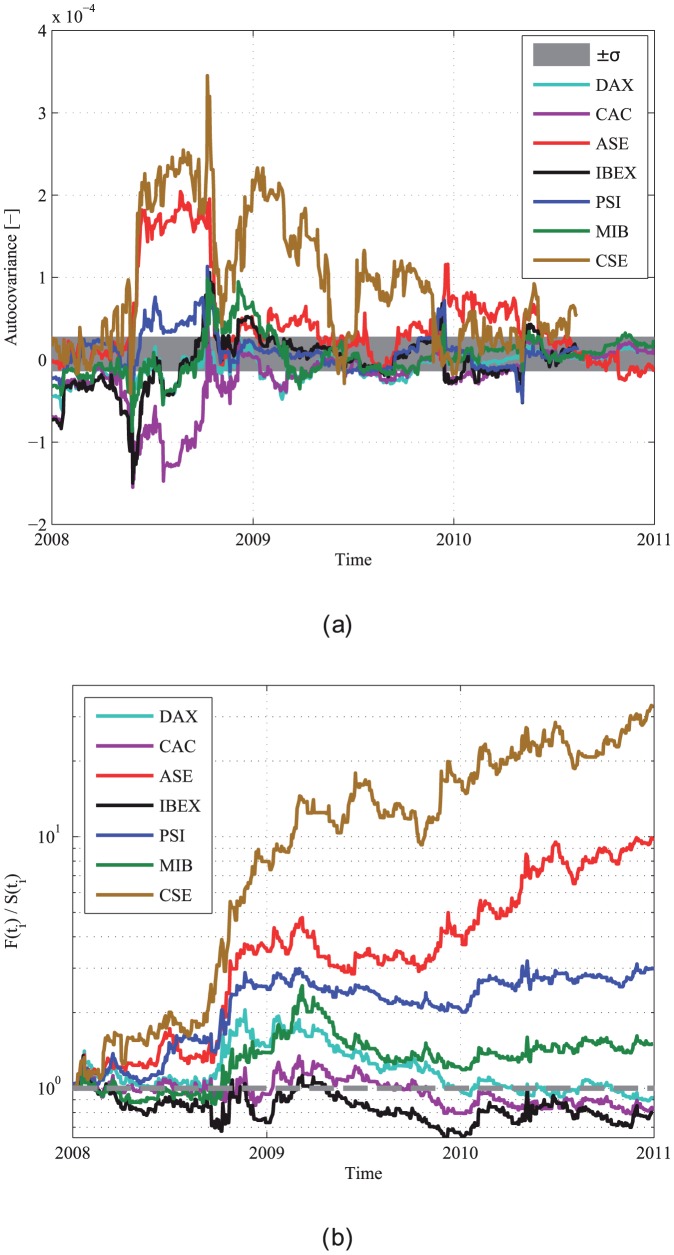
The autocovariance and the RA results for several major European markets in the period 2008–2011. (a) The autocovariance of the German DAX index (cyan), the French CAC index (magenta), the Greek ASE index (red), the Spanish IBEX index (black), the Portuguese PSI index (blue), the Italian MIB index (green) and the Cypriot CSE general index (brown). The gray area marks one standard deviation of the autocovariance from 0, estimated by data shuffling. The calculations were performed using a 100 days long sliding window; (b) The RA results for several major European stock market indices in the period 2008–2011. The ratio 1 is marked by a dashed gray curve.

The analysis techniques introduced can also be used to compare between the reaction of different European stock markets to the global financial crisis and the European debt crisis, using the insights of the comparison between developed and emerging markets. Fig. 6 presents the results of the autocovariance and the RA profitability ratio for several major European markets between 2008 and 2011. Similarly to the results presented in Figs. 3 and 4, two behaviors or regimes can be identified - positively autocorrelated dynamics with a large RA profitability ratio and vice versa. The Portuguese, Greek and Cypriot stock markets show results that resemble the results obtained for emerging markets such as the Russian and the Indian markets, with significant positive autocovariance (using the runs test, 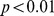). On the other hand, most western European markets are prominent examples of developed markets. The behavior of the Italian market cannot be solely identified with one of the regimes. This division coincides with the well known effects of the recent crises on the European economies - as Greece, Portugal and Cyprus were extremely effected by the crises, together with Spain and Italy, which were less effected. France and Germany are considered to be the major European markets least effected by the recent crises [37]. Moreover, Greece was officially degraded from being a developed economy to an emerging market [38].

These results once again demonstrate that the autocovariance is a measure of the market's intricacy and development status. Moreover, the reaction of the autocovariance to financial crises can also be connected to the flexibility and stability of a market, so that the autocovariance can serve as a measure for the invulnerability of the market.

## Discussion

The 2007–2009 global financial crisis brought more attention to the validity of the current economic theories, and specifically renewed a debate regarding market predictability and possible forecasting of asset prices [39]–[41]. Treating the dynamics of security prices as a Brownian motion contradicts the idea of speculative bubbles, which many financiers and economists see as one of the major causes of the crisis [41]–[44]. Therefore, new methods of analysis should be presented in order to test the conventional theories and hypotheses. Here we presented a method of analyzing the autocovariance of assets, specifically indices, which extracts temporal order found in the index price returns. This method consisted of calculating the distribution of the autocovariance of an index daily closing price using sliding windows of different lengths. Suggesting that major deviations from a random series exist, an algorithm, which is capable of exposing these deviations, was described and investigated. The success of this algorithm is strongly correlated with the autocovariance, and can be seen as a simple way to unveil autocovariance which is above or below zero.

It was found that some of the major indices demonstrate considerable deviations from a random behavior in terms of their average autocovariance (see Figs. 1 and 2). These results might also indicate a degree of inefficiency in market indices. The efficient market hypothesis (EMH) is a key notion in modern finance, and has been the subject of major debates in the past few decades. The basic principle behind the EMH is that asset prices reflect all available information regarding these assets at any time [45]. According to the weak form of EMH [45], markets should follow a Markov process. Therefore, a major deviation from their Markovity is an indicator of inefficiency. Moreover, the average autocovariance of different stocks and indices should tend to zero according to the same logic. The results of the conducted analysis can therefore imply the inefficiency of several major indices. Consequently, the market weak form efficiency, which is an underlying assumption of many popular financial models [1]–[3], [17], should be taken into account cautiously, most notably during periods of market stress [34].

Emerging and developed markets were compared in terms of their autocovariance for the period of 2000–2013. It was found that a fundamental difference exists between these types of markets. Emerging markets showed positive average autocovariance values with a positive spike during the global financial crisis, where developed markets showed the opposite behavior during the same period. These results join previous works that pointed out fundamental differences between emerging and developed markets in a variety of properties [28], [46]–[50]. The following are possible sources of the observed differences:

- Algorithmic trading became highly influential in the past decade in developed markets, while for emerging markets, it is still not as dominant. It is generally thought to increase market efficiency [51], [52]. In addition, the derivative market and ETF trading are much more advanced in developed markets than in emerging economies [53]. These financial instruments has a general effect of increasing the market efficiency, indicating a reduce in return predictability [54].
- In most emerging markets, the past few decades are a transition period from planned or centralized economy to competitive market economy. This transition is usually fraught with market concentration that reduces efficiency [55].
- In terms of capitalization, emerging markets are usually smaller, compared to developed markets and the trading volumes in these markets are much smaller than in developed markets per capita [56]. Empirical evidences were found to relate relatively low trading volumes and market inefficiency [4], [57], [58]. This, however, should be treated with caution, as some of the most stable markets in the world are relatively small.

An important result in this context, is the uniqueness of the Chinese market. The Chinese market was found to behave as in an average state between a developed and an emerging market in terms of the dynamics of its autocovariance (see Fig. 4), which might be credited to its size (when compared to Russia and Mexico, for example) on one hand and to its state of development (when compared to India) on the other hand.

The US stock market that was analyzed for 1950–2010, have shown a transition between a long period of significantly positive autocovariance during 1950–1980, to a more intricate and random-like behavior from 1980 onward. This transition is an indication for maturing processes that possibly characterized the developed economies during the past decades, while the less developed markets might not went through yet.

In addition, developed and emerging markets show different reaction to crises. As the autocovariance tends to decrease following a crisis in developed markets, it increases in emerging markets. This argument was also supported by analyzing the reaction of European stock markets to the recent financial crises. The reaction of the less developed economies of Europe, that were also severely effected by the crises, notably Greece, Portugal and Cyprus, showed similar results to those obtained for emerging markets. These results show that the presented analysis tools can be used for evaluating the development of an economy and its market degree of perfection and intricacy. In addition, the classification to developed and emerging markets using the autocovariance framework, and to the two identified regimes, can be used not only as an indicator for the market degree of development, but as a predictor for its reaction to financial crises. Looking at the results for the European markets during 2008–2011, economies with a more sophisticated and intricate stock market, that exhibit relatively low or no autocovariance, were likely to be less effected by the economic crises and in that sense can be regarded as more stable.

Taking the interpretation of the results a step further, we can conclude that economies with efficient stock markets that are characterized by neither substantial positive nor negative autocovariance, are likely to be less vulnerable to crises and more flexible in times of stress. Therefore, regulators and policy makers should act to reduce inefficiency. However, this interpretation needs to be further explored.

Establishing the above, more research should be carried out: The characterization of the RA in terms of other financial, economic and statistical properties other than the autocovariance; deeper investigation of the connection between the dynamics of autocovariance and periods of financial stress and market crashes; applying the analysis methods used to intraday market data; A thorough historical analysis of the autocovariance dynamics of major markets during the past century; further investigation of emerging markets and indicators of their inefficiency, and determining the factors that contribute to autocovariance in the stock market.

## Methods

### Data retrieval

We analyze data sets of the daily index values of major international stock market indices. We retrieved this data from Yahoo! Finance (http://finance.yahoo.com/) and from the Bloomberg database (http://www.bloomberg.com/markets/stocks/world-indices/).

### The derivation of an unbiased autocovariance expression

Eq. (1) presents an expression used for calculating the autocovariance of a given time series. However, this expression is biased, as the sample means are taken into account. Here we derive an unbiased expression for calculating this term. It should be noted, that this derivation is done for a stationary process, with constant mean and standard deviation values. Since real financial data is not stationary, only periods of up to 100 days will be taken into account, in order to avoid the distortion of the data, as for such periods, the changes are relatively mild.

Let us denote the real autocovariance of a time series as 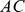, and the calculated sample autocovariance as 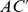. 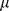 and 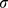 denote the real mean and standard deviation, respectively, where 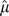 denotes the sample mean. In addition, we use 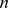 to denote the sample length and assume that 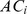 is the autocovariance for a lag of 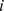 days, and denote 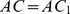. If we take the expectation value of the definition in Eq. (1), we get:
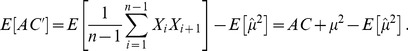
(3)


We should now evaluate 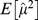:
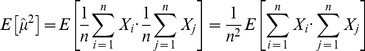
(4)

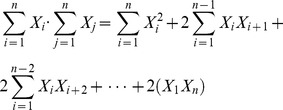
(5)


Therefore, we can deduce that:
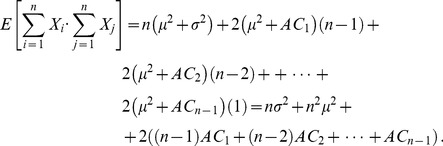
(6)


Under the assumption that the autocovariance decreases rapidly with the lag, we omit 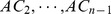 and obtain:
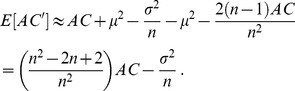
(7)


Therefore, in order to calculate an unbiased expression for the autocovariance we should take:
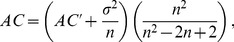
(8)which is the expression presented in Eq. (2).

### Statistical analysis

The deviations of the autocovariance series calculated for real market data from an autocovariance series derived from a random series were evaluated using a runs test, which is a simplification of the Wald-Wolfowitz runs test [27]: For a given time series of length 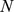, we calculate its corresponding autocovariance series, 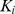, of length 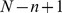, with a sliding window of length 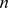. Following this, we count the data points in which 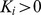, denoted by 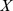, and define 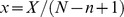. For an autocovariance series derived from a random series, using a sliding window of 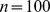 days, 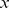 is normally distributed with 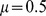 and 
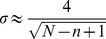
. Since this distribution is normal, for any given real autocovariance series we can calculate the Z-score 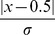, for the appropriate value of 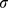, and derive the *p*-value of the test. This will enable us to reject the null hypothesis that the given autocovariance series is similar to an autocovariance series derived from a random data set if the resulting *p*-value is sufficiently low (lower than 0.01).

We used the autocovariance as the main statistical measure analyzed in this work. The autocorrelation, equal to the autocovariance normalized by the variance of the data set, can be also used. Since these two properties differ only in their absolute value, the conclusions drawn from the presented results will hold when the autocorrelation is analyzed instead of the autocovariance, as the standard score of the runs test is equal for both statistical measures. However, presenting the results in terms of the autocovariance enables a better understanding of the analysis and its implications, without effecting the statistical significance of the results. In addition, the derivation of the anti-biasing correction term for the autocovariance cannot be simply applied to the autocorrelation, which might result in a substantial biasing of the autocorrelation results.

## Supporting Information

Datalinks S1 **Hyperlinks to the online sources of the data used in the paper are provided.**
(PDF)
