# Supplementary material for: Unraveling Hidden Order in the Dynamics of Developed and Emerging Markets
Source: PLoS One. 2014 Nov 10;9(11):e112427. doi: 10.1371/journal.pone.0112427 (PMC4226548; doi:10.1371/journal.pone.0112427)
Supplement: Datalinks S1 — Hyperlinks to the online sources of the data used in the paper are provided. (PDF) [file pone.0112427.s001.pdf]

Datalinks – S1

Below are the hyperlinks to the online sources of the data used in the paper:

1. BSE – Bombay SENSEX Index – http://finance.yahoo.com/q/hp?s=%5EBSESN+Historical+Prices
2. CSE – Cyprus Stock Exchange – http://www.bloomberg.com/quote/CYSMMAPA:IND
3. DJI – Dow Jones Industrial Index – http://www.bloomberg.com/quote/INDU:IND
4. FCHI – CAC French Index – http://finance.yahoo.com/q/hp?s=%5EFCHI+Historical+Prices
5. FTSE – FTSE100 British Index – http://finance.yahoo.com/q/hp?s=%5EFTSE+Historical+Prices
6. GDAXI – DAX30 German Index – http://finance.yahoo.com/q/hp?s=%5EGDAXI+Historical+Prices
7. GSPC – S&P500 American Index – http://finance.yahoo.com/q/hp?s=%5EGSPC+Historical+Prices
8. HSI – Hang Seng Index (Hong Kong) – http://finance.yahoo.com/q/hp?s=%5EHSI+Historical+Prices
9. IBEX – IBEX Spanish Index – http://finance.yahoo.com/q/hp?s=%5EIBEX+Historical+Prices
10. IPC – IPC Mexican Index – http://finance.yahoo.com/q/hp?s=%5EMXX+Historical+Prices
11. IXIC – NASDAQ Index – http://finance.yahoo.com/q/hp?s=%5EIXIC+Historical+Prices
12. MIB – Milano Stock Exchange – http://finance.yahoo.com/q/hp?s=FTSEMIB.MI+Historical+Prices
13. N225 – NIKKEI Index (Japan) – http://finance.yahoo.com/q/hp?s=%5EN225+Historical+Prices
14. PSI – Portuguese Stock Index – http://www.bloomberg.com/quote/PSI20:IND
15. RTSI – RTS Russian Index – http://finance.yahoo.com/q/hp?s=RTS.RS+Historical+Prices
16. SSE – Shanghai Stock Exchange (China) – http://finance.yahoo.com/q/hp?s=000001.SS+Historical+Prices
17. TA100 – Tel Aviv 100 Index – http://www.bloomberg.com/quote/TA–100:IND
18. TA25 – Tel Aviv 25 Index – http://www.bloomberg.com/quote/TA–25:IND
